# Structures and biological functions of zinc finger proteins and their roles in hepatocellular carcinoma

**DOI:** 10.1186/s40364-021-00345-1

**Published:** 2022-01-09

**Authors:** Xinxin Li, Mengzhen Han, Hongwei Zhang, Furong Liu, Yonglong Pan, Jinghan Zhu, Zhibin Liao, Xiaoping Chen, Bixiang Zhang

**Affiliations:** 1 Hepatic Surgery Center, Tongji Hospital, Tongji Medical College, Huazhong University of Science and Technology, 1095 Jiefang Avenue, Wuhan, 430030 China; 2 Hubei Key Laboratory of Hepato-Pancreato-Biliary Diseases, Wuhan, 430030 China

**Keywords:** Zinc finger protein, Transcription factor, Hepatocellular carcinoma, Transcription regulation, Biological function

## Abstract

Zinc finger proteins are transcription factors with the finger domain, which plays a significant role in gene regulation. As the largest family of transcription factors in the human genome, zinc finger (ZNF) proteins are characterized by their different DNA binding motifs, such as C2H2 and Gag knuckle. Different kinds of zinc finger motifs exhibit a wide variety of biological functions. Zinc finger proteins have been reported in various diseases, especially in several cancers. Hepatocellular carcinoma (HCC) is the third leading cause of cancer-associated death worldwide, especially in China. Most of HCC patients have suffered from hepatitis B virus (HBV) and hepatitis C virus (HCV) injection for a long time. Although the surgical operation of HCC has been extremely developed, the prognosis of HCC is still very poor, and the underlying mechanisms in HCC tumorigenesis are still not completely understood. Here, we summarize multiple functions and recent research of zinc finger proteins in HCC tumorigenesis and progression. We also discuss the significance of zinc finger proteins in HCC diagnosis and prognostic evaluation.

## Background

Zinc finger proteins (ZFPs), which constitute the largest transcription factor family with finger-like DNA binding do- mains, play a significant role in multiple biological processes. ZFPs primarily function as transcription factors in tumorigenesis and tumor progression. Transcription factors (TFs) are proteins that play a vital role in complicated biological processes, such as metabolism, autophagy, apoptosis, immune responses, stemness maintenance and differentiation. TFs regulate transcription of genes by recognizing or binding to DNA sequences directly [1, 2]. So far, zinc finger motifs have been classified into eight different categories according to their main-chain conformation and secondary structure around their zinc-binding sites, including Cys2His2 (C2H2) like, Zn2/Cys6, Treble clef, Zinc ribbon, Gag knuckle, TAZ2 domain like, Zinc binding loops and Metallothionein [3, 4]. In addition to these zinc motifs, ZFPs also contain several domains that play different roles in cell biological processes, including BTB (Broad-Complex, Tramtrack, and Bric-a-brac), the Krüppel-Associated Box (KRAB) domain, SET domain and SCAN (SRE-ZBP, CTfin51, AW-1 and Number 18 cDNA) domain. Because of the diversity of zinc finger motifs and these domains, ZFPs can play different roles in gene regulation under various cellular environments and other stimuli.

Hepatocellular carcinoma (HCC) accounts for a major part of the global cancer category. It accounts for approximately 75–80% of primary liver cancer [5, 6]. According to the Global online database of infectious diseases analysis, the incidence of liver cancer decreased in many Asian countries during 1978 ~ 2012. Still, it rebounded in India, the United States and other European countries [6]. Since patients have no obvious clinical symptoms in the early stage of HCC, the early diagnosis of HCC becomes extremely difficult. Although there are many treatments, including surgical resection, chemotherapy, targeted therapy, the prognosis of liver cancer is still not optimistic. However, the numerous molecular mechanisms in HCC pathogenesis remain unclear. In recent years, studies about ZFPs functions in multiple cancers have been constantly emerging [7–14]. These studies offered promising treatments for malignancies, including HCC [15]. In this review, we will discuss ZFPs basic spatial structures and the complex mechanisms of ZFPs in HCC.

## Structures of zinc finger proteins

### BTB domain

The BTB domain (also known as the POZ domain) is a multifaceted protein-protein interaction motif found in whole eukaryotes. At the same time, it has been reported in previous literature that some poxvirus proteins are similar to a part of ZFPs, like ZID, GagA and ZF5; this domain has been named as POZ (poxvirus and zinc finger) domain [16, 17]. As a highly conserved structure, it engaged in multiple cellular functions, including transcription repression, cytoskeleton dynamics, tetramerization and gating of ion channels, and targeting proteins for ubiquitination [18–23]. The BTB domain consists of a cluster of five α-helixes, one end covered by a short three-chain β-fold, which is compact and spherical [24]. In different BTB domains, their primary structure is less conservative, but their secondary structure is broadly similar. It has been reported that the BTB domain can be classified into four families: T1, Skp1, ElonginC, and BTB-ZF, but the details have not yet been worked out. Interestingly, the BTB domain in ZFPs can be homologous and heterogenous, or the BTB domain can recruit co-inhibitors of transcription. The function of proteins containing the BTB domain can be roughly divided into two categories: transcriptional inhibition and protein degradation, which are crucial for genes to function in cell development [25–27]..

### SCAN domain

Scan domain, a highly conservative domain, consists of 84 amino acids rich in leucine residues, is also known as the leucine-rich region [28, 29]. To date, about 244 protein products containing the scan domain have been identified in the human genome, of which about 50 scan-containing transcription factors have been described. The scan domain has an amphipathic secondary structure that participates in protein-protein interaction, especially self-binding and mediated oligomerization [25, 30, 31]. Further studies of the domain have shown that it can interact with separated scan domains, such as scan domain protein 1 (SDP1), or with other family members with scan domains [32]. It is worth mentioning that this interaction is not universal but selective, suggesting that not all family members can form oligomerization. Interactions between different scan-containing transcription factors lead to various transcription activities. In addition, few proteins may have several special names depending on their domain. As can be seen from the NCBI gene database, the human ZSCAN (zinc finger and scan) transcription factor family members have a uniform name ranging from ZSCAN1 to ZSCAN54 [33].

### KRAB domain

**Fig. 1:**
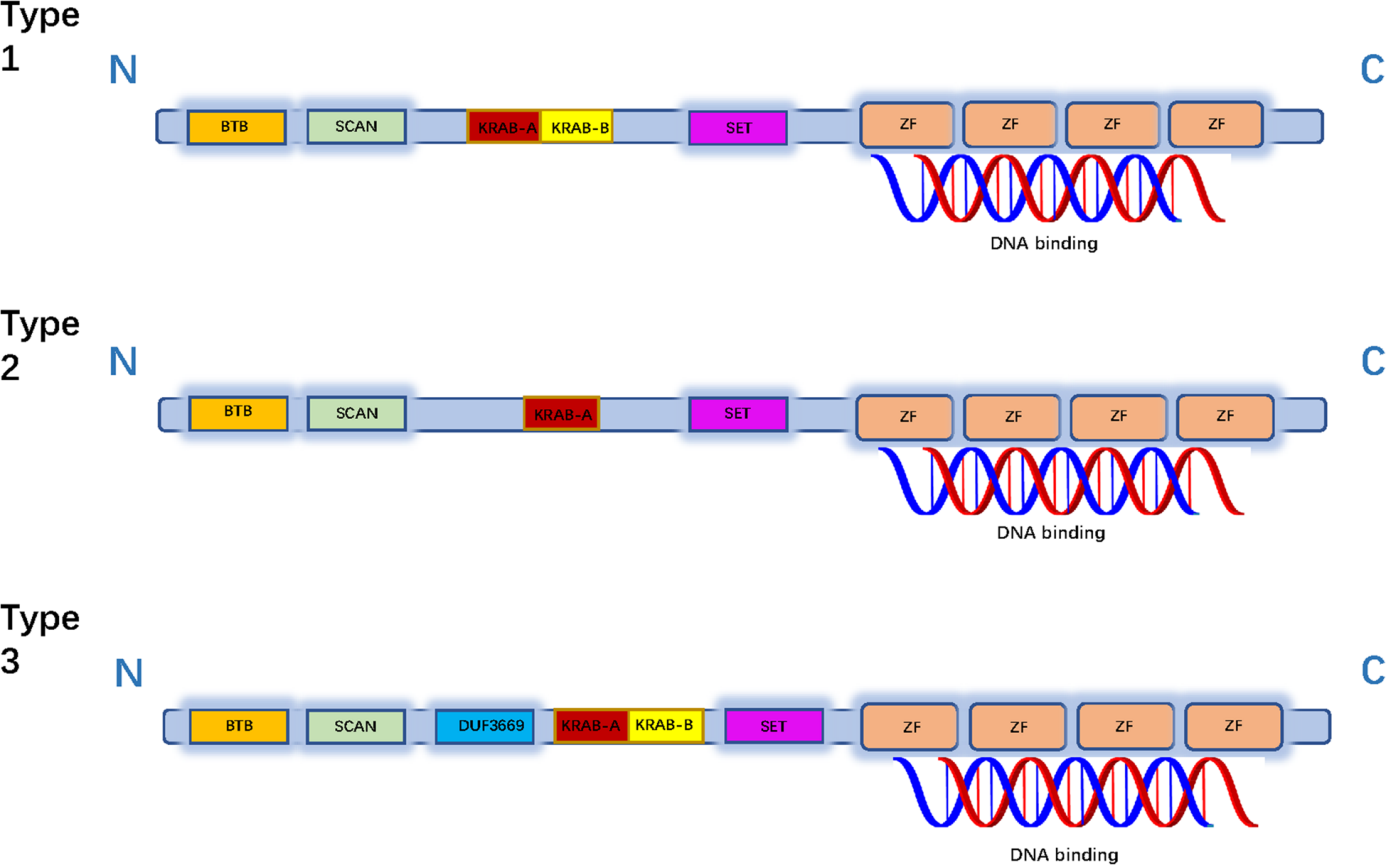
Several domain structures of C2H2-ZFPs. Three diverse forms of C2H2-ZFPs are described. Each C2H2-ZFP contains at least one KRAB structural domain, BTB domain, SCAN domain, SET domain and several zinc fingers which can bind to DNA sequences. In brief, the KRAB domain can be divided into two parts: the A-box (KRAB-A) and the B-box (KRAB-B). As shown in type 4, some C2H2-ZFPs contain a DUF3669 domain. Functionally, BTB domain is mainly responsible for transcriptional repression and protein degradation; SCAN domain is mainly responsible for protein binding and protein oligomerization; KRAB domain is mainly responsible for repression of transposable elements; SET domain is mainly responsible for protein methylation (mainly histones); C2H2 motif can bind to DNA, RNA, and proteins to perform different functions, but most of them bind to DNA.ZF: zinc finger; BTB: Broad-Complex, Tramtrack, and Bric-a-brac. KRAB:Krüppel-associated box; SCAN: SRE-ZBP, CTfin51, AW-1, and Number 18 cDNA; DUF3669:domain of unknown function 3669

Krüppel-associated box (KRAB) domain-containing zinc finger proteins (KZFPs), which have been reported to exist only in quadrupeds. The human genome encodes approximately more than 350 KZFPS. In recent reports, KZFPs mainly inhibit transposable elements (TEs) by recruiting transcriptional regulators and heterochromatin formation and DNA methylation in embryonic stem (ES) cells [34, 35]. The KRAB domain consists of 75 amino acids, and the domain is usually disconnected into two adjoining modules: A-box, which is mainly responsible for inhibiting activity by interacting with corepressors; and B-box, which is believed to augment the ability of repression of A-box through some undiscovered mechanisms [36–38]. Theoretically, the length of KRAB-ZFP should be enough to recognize longer DNA target sequences specifically, but in fact, the binding motif of KRAB-ZFP is often shorter than predicted. This implied that in KRAB-ZFP, different ZNF recognize different DNA motifs [39]. In addition, ZNF is not only involved in contacting with DNA but also can participate in other categorizes of interactions, such as interactions with RNA, or proteins [40–43]. A few ZFPs contain added domains, including BTB domain, SCAN domain, KRAB domain, SET domain, DUF3669 domain and C2H2 motif. Regrettably, the precise function of the DUF3669 domain is still unclear (Fig. 1).

### SET domain

The SET structural domain is widely found in eukaryotes and consists of approximately 130 amino acids and is named after the Drosophila proteins Suppressor of variegation 3–9 (Su (var)3–9), Enhancer of zeste (E(z)), and Trithorax (Trx). The biological behaviours in which the SET structural domain is involved are primarily associated with the methylation of substrates. For example, many SET domain-containing proteins can mono-, di- or trimethylation of their lysine substrates using the cofactor S-adenosyl-L-methionine (SAM), these catalyzed targets include histones and many non-histone substrates. In addition, the SET domain does not usually exist as a stand-alone entity, and in many proteins, it co-exists with other protein structural domains and interacts with proteins that regulate its catalytic function [44, 45].

### C2H2-zinc finger motif

Among eight different groups of zinc fingers, the most classical zinc finger is the C2H2-like finger [3]. As the most prevalent motif in ZFPs, over 5000 C2H2-like fingers have been encoded by the human genome [46]. The zinc finger structure is generally located at the C-terminus of the ZFPs. The consensus sequence for C2H2 zinc fingers is CX2CX3FX5LX2HX3H, which contains conservative hydrophobic residues wrapped in hydrophobic cores except for its two cysteines and two histidine residues. These conservative amino acids and a characteristic structure is consisting of a two-stranded antiparallel β-sheet and an α-helix [47]. It has been suggested in the article that an individual zinc finger motif binds an adjacent three-nucleotide subsequence, maybe three to five, and a C2H2-zinc finger domain can be specified to a range of three base pair (3 bp) targets [48, 49].

C2H2-type ZFPs generally contain added anatomic domains, such as BTB (Broad-Complex, Tramtrack, and Bric-a-brac), the Krüppel-Associated Box (KRAB) domain, SET domain and SCAN (SRE-ZBP, CTfin51, AW-1 and Number 18 cDNA) domain [40]. These structures regulate immune response, cell differentiation and embryonic development at the transcription and translation level through specifically binding to the target molecule DNA, RNA, DNA-RNA sequence, and binding to itself or other ZFPs [48]. For instance, ZEB1 can actuate transcription through binding to coactivators, such as p300 and P/CAF [50, 51].

### Comparison of the functions between these structural domains

The domains of ZFPs are not only diverse, but the functions of these domains are also very distinct. For example, although both the BTB domain and the SCAN domain can bind to the protein, but the BTB domain mostly inhibits the transcriptional activity of the target gene by recruiting transcription co-repressors, while the SCAN domain causes oligomerization of target proteins through its specific binding [27, 31]. In addition, KZFPs can inhibit transposable elements through DNA methylation, while SET domains that have different substrates are through protein methylation to play its catalytic role [34, 44]. In a word, various C2H2-type ZFPs contain different types of the above-mentioned domains, and these ZFPs with different functions play a significant role in in cell biological processes.

## Biological functions of zinc finger proteins

Among the numerous biological functions of ZFPs, transcription regulation occupies a highly prominent position. ZFP relies on the domain-containing zinc finger in one segment to bind to the promoter region of the target gene and act as a transcription enhancer or transcription inhibitor. In addition to transcriptional regulation, ZFPs can induce protein-protein interactions, bind to RNA, and interact with proteins and RNAs in the meantime [52–55].

### Transcription regulation

As the most prominent role of ZFPs, the functions of transcriptional regulation are mainly reflected in both transcriptional promotion and transcriptional repression. It has been recently reported that ZEB1 (Zinc finger E-box binding homeobox 1) can bind to the HDGF (hepatoma-derived growth factor) promoter to stimulate HDGF transcription in EC (endometrial carcinoma) cells [56]. Another example on the contrary, ZNF322A, a C2H2 zinc finger transcription factor, can directly bind and recruit histone deacetylation enzyme 3 to the c-Myc promoter to suppress c-Myc expression at the level of transcription. Then increase mitochondrial phosphorylation to promote cell movement and ultimately maintain stem cell-like characteristics in lung cancer [57].

### Protein interactions

As well as functioning as transcription factors, numbers of ZFPs that can induce protein interactions have also been identified in recent years [58]. For example, the striated muscle RING zinc finger protein (SMRZ) is a novel human striated muscle ZFP which has a ring domain at its N-terminal. In recent studies, the SMRZ was reported to interact with SMT3b, which was a ubiquitin-like protein, and their interactions are completed by the ring domain of SMRZ. This kind of interaction may contribute to the regulation of cell cycle that occurs during the process of growth in striated muscle cells [59].

### Post-transcriptional regulation

Some ZFPs also have RNA binding properties, and these ZFPs are generally thought to be involved in post-transcriptional regulatory processes such as mRNA maturation splicing and degradation [60, 61]. Recently, the zinc finger protein Regnase-1, which had been revealed to regulate self-renewal of HSPCs (Hematopoietic Stem and Progenitor Cells) through modulating mRNA stability. More specifically, Regnase-1 could degrade the mRNAs of Gata2 and Tal1 by targeting the 3’UTR region of their mRNAs, and caused a decrease in the expression of GATA2 and Tal1, which could ultimately lead to a slowdown in the self-renewal of HSPCs and affected the homeostasis of HSPCs [62].

### Effects of ZFPs via different mechanisms

#### Lipid metabolism

An increasing number of studies have shown that metabolic manipulate and signaling pathways are closely linked, not only in normal cells but also in cancer cells [63]. Recently, Li et al. suggested that YY1 prevented proliferator-activated receptor gamma coactivator-1β (PGC-1β) expression by directly binding to its promoter, thereby inhibiting the oxidation of fatty acid β, which led to the accumulation of lipid in HCC cells and induced the carcinogenic potentiality of HCC cells. It was worth mentioning that the reduction of the PGC-1β expression level by YY1 was independent of HIF-1α (hypoxia-inducible factor-1α) expression. This provided us with new insights into the conditions under which HIF-1α works [64–66]. Liu et al. revealed that ZBTB20, a regulator of lipid homeostasis, promoted hepatic de novo lipogenesis (DNL) by directly binding to and enhancing the activity of the ChREBP-α promoter and indirectly activating ChREBP-β, which provided new insights into the transcriptional regulatory network of DNL and had the potential to be a target for the treatment of fatty liver disease (FLD) [67].

#### Cell differentiation

ZFPs like Snails (Snai1, Snai2/Slug, Snai3/Smuc) and the E-box binding proteins ZEBs (ZEB1, ZEB2) can also affect the process of cell differentiation directly or indirectly [68]. Goossens et al. demonstrated that ZEB2 had high mRNA levels in hematopoietic stem cells and hematopoietic progenitor cells (HPCs). Sophisticated cellular analysis showed that ZEB2 was imperative for normal HSC/HPC differentiation, and ZEB2 deficient HSCs/HPCs were not properly implanted in fetal liver or bone marrow, and exhibited boosted adhesion associated with upregulated expression of beta1 integrator and CXCR4 [69–74]. Tang et al. expounded that Snail and Slug could cooperatively control skeletal stem/stromal cell (SSC) self-renewal in knockout mouse models. Mechanistically, Snail/Slug were reported to regulate SSC function by forming a complex with transcriptional co-activators YAP and TAZ, thereby inhibiting the Hippo pathway-dependent YAP/TAZ signaling cascade regulation. The regulatory network within the above mechanism provides new thought on cell differentiation in normal hepatocytes and HCC cells [75]. In addition, snail can also be involved in regulating cell stemness in glioma stem cells (GSCs). A recent study revealed that snail could inhibit TGFβ1 transcription and reduce its activity through a positive feedback loop resulting from its interaction with SMAD. This process contributes to regulation of the opposite BMP and TGFβ pathway activity, thereby inhibiting GSC stemness ultimately [76].

#### Immune response

Few ZFPs have recently been reported in immune-related processes such as immune response, immune homeostasis, and cytokine production in emerged new studies [77, 78].

Gfi1, which function as a transcriptional repressor, is a zinc finger protein that participates in diverse development contexts. Jin et al. demonstrated that Gfi1 (growth factor independent 1 transcriptional repressor) played a limiting role in the inflammatory response induced by endotoxin in mouse lung, and its regulatory function in alveolar macrophages as downstream of LPS (Lipopolysaccharide) receptor (TLR4) and upstream of TNF further elucidated the mechanism of Gfi1’s role in the endotoxin response [79]. By the way, the toll-like receptor (TLR) family plays a directive role in inflammatory response, antiviral, and activation of transcription factors [80]. Moreover, ZFYVE1 (zinc finger FYVE-type containing 1) can bind to poly(I:C) and TLR3 (Toll-like receptor 3) through the FYVE structural domain and enhance its association with TLR3 in response to poly(I:C) stimulation. Subsequent overexpression of ZFYVE1 can also significantly promote the binding of TLR3 to its ligand poly(I:C). Although the absence of ZFYVE1 can inhibit TLR3-mediated innate immune and inflammatory responses, it cannot inhibit TLR4-mediated the same reactions as TLR3 [81]. The above roles of these ZFPs in innate immunity and the associated inflammatory response provide deep thinking for subsequent studies.

CCCH zinc finger proteins consist of several CCCH zinc finger domains, which are composed of one histidine and three cysteines. A small number of CCCH zinc finger proteins have been reported to play important roles in immune responses [82, 83]. For instance, TTP (aka ZFP36), roquin 1 and MCPIP1, can form a regulatory network that maintains immune homeostasis [84–86]. The regulatory network composed of these three ZFPs accelerates the regression of inflammation, manage the sizes and rhythms of the adaptive immune response, targets mRNA to regulate its half-life and regulates signal pathways [87, 88]. However, based on the characteristics of this type of ZFP that can shuttle between different cell compartments, there is still a lot to be studied, especially in cell metabolism and cellular immunity [89].

## Zinc finger proteins in HCC

**Fig. 2:**
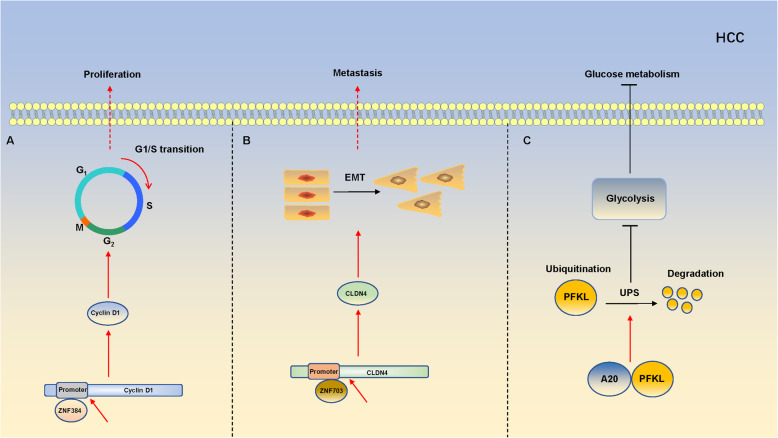
Zinc finger proteins influence hepatocarcinogenesis through different ways. **A**: ZNF384 activates Cyclin D1 transcription by directly binding to its promoter, and facilitates G1/S phase transition, ultimately promotes proliferation of HCC. **B**: ZNF703 promotes HCC metastasis and induces through transactivating CLDN4 expression. **C**: A20 decreases the protein level of PFKL by promoting ubiquitination and degradation of PFKL, then inhibits progression of HCC through downregulating glycolysis

In the past decades, the role of ZFPs has been reported in various of cancers including nasopharynx, esophagus, lung, gastric, colorectal, breast, thyroid, prostate, ovarian cancer [57, 90–97]. However, the number of studies in HCC is much less than other cancer types, and there are very few systematic reviews about HCC [98–101]. To provide a better understanding of ZFPs in HCC and explore the potential therapeutic targets. Here, we firstly summarize the functions of ZFPs in HCC and the specific mechanisms (Tables 1 and 2), and some specific examples are shown in the form of figure (Fig. 2).

**Table 1:** Oncogenic role of ZFPs in HCC

ZFPs	Aliases	Role	Target genes	Mechanism in HCC	Ref
ZNF384	NMP4、CAGH1	Oncogene	Cyclin D1	Promotes proliferation	[102]
ZNF263	ZSCAN44、FPM315	Oncogene	Beclin1、LC3	Promotes proliferation, chemotherapy resistance and inhibits apoptosis	[103]
ZNF703	NLZ1、ZPO1	Oncogene	CLDN4	Induces EMT progress	[104]
ZNF687	PDB6	Oncogene	BMI1、OCT4、Nanog	Enhances invasion and chemoresistance	[7]
ZNF143	SBF、STAF	Oncogene	MDIG	Promotes proliferation and tumor growth	[101]
ZNF191	ZNF24、ZSCAN3	Oncogene	CTNNB1	Activates Wnt signaling pathway	[105]
ZNF503	Nlz2、NOLZ1	Oncogene	GATA3	Promotes migration, invasion and EMT progress	[106]
ZFX	ZNF926	Oncogene	Nanog、SOX-2	Enhances proliferation, drug resistance, and the ability of self-renewal	[107]
Gli1	PPD1、PAPA8	Oncogene	MMP-2、MMP-9	Promotes migration and invasion	[8]
			Caveolin-1	Induces EMT and promotes the motility and invasion	[9]
			Twist	Induces EMT progress	[10]

**Table 2:** Tumor-suppressive roles of ZFPs in HCC

ZFPs	Aliases	Role	Target genes	Mechanism in HCC	Ref
A20	TNFA1P2	TSG	Twist1	Inhibits proliferation and migration	[98]
			FAK、RAC1	Inhibits the motility and metastasis	[11]
			PFKL	Inhibits proliferation, migration and glycolysis	[108]
GATA4	TOF、ASD2	TSG	NF-κB1, NF-κB2, RELA	Induces the MET transition	[109]
ZNF307	ZKSCAN4	TSG	Caspase-3、BAX、BCL-2	Inhibits proliferation	[110]
ZNF191	ZNF24、ZSCAN3	TSG	DLG1	Inhibits metastasis of HCC	[111]
ZFP91	PZF、ZNF757	TSG	HNRNPA1	Inhibits glucose metabolic reprogramming, proliferation and metastasis	[112]
ZNF382	KS1	TSG	Fos、Jun、DVL2、FZD1	Activates the p53 signaling pathway	[12]
ZNF774		TSG	NOTCH2	Inhibits growth and metastasis of HCC	[13]
Miz1	ZBTB17、ZNF60	TSG	MTDH	Inhibits inflammation	[113]
ZNF521	EHZF、Evi3	TSG	Runx2	Inhibits growth	[14]

### Cell cycle

The cell cycle regulation cannot be separated from cyclins, CDKs (Cyclin-dependent kinases) and CKIs (Cyclin-dependent kinase inhibitors) [114]. CDC6 (cell division cycle 6) is a protein essential for the initiation of DNA replication [102]. The overexpression of ZNF143 facilitated HCC cell cycle progression via activating CDC6. Concretely, ZNF143 was reported to directly activate transcription of histone demethylase mineral dust-induced gene (MDIG) and decreased the enrichment of H3K9me3 in the CDC6 promoter region [115]. In addition, Cyclin D1 could form a complex with CDK4 or CDK6, whose activity was required for cell cycle progression [116]. He et al. demonstrated that knockdown of ZNF384 could result in sluggish of the G1/S phase transition in HCC. Mechanistically, ZNF384 was found to upregulate Cyclin D1 by binding to its promoter region, then accelerated G1/S phase transition and promotes proliferation of HCC [110].

### Apoptosis

The process of apoptosis is induced by two distinct pathways: the intracellular pathway or the extracellular pathway [117]. In the extracellular pathway, apoptosis begins with binding a family of death receptors to an appropriate ligand on the membrane, then the death-inducing signaling complex (DISC) is recruited and activated, which consists of FADD (Fas-Associated protein with Death Domain), the death-executing protease enzyme caspase-8 and FLIP [118]. Liang et al. revealed that overexpression of ZNF307 upregulated the protein level of caspase-3 and Bax (BCL2 associated X), while decreased the protein level of Bcl-2 (BCL2 apoptosis regulator). It was the first time that ZNF307 was reported to function as tumor suppressor in HCC by inducing apoptosis by targeting these genes [103]. It was worth mentioning that Bax and Bcl-2 is also regulated by other ZFP in HCC. In a previous study, GLI1 was aberrantly overexpressed in HCC [119]. PCAF modulated the GLI1/Bcl-2/BAX axis to induce apoptosis in HCC. Gai et al. suggested that the cytoplasmic GLI1 protein was acetylated directly at lysine 518 by PCAF (P300/CBP-associated factor), while the nuclear translocation and promoter occupancy of GLI1 were prevented. Subsequently, the expression of Bcl-2 was decreased, and BAX was increased [120]. Additionally, ZNF263 could promote resistance to apoptosis in HCC indirectly by activating ER stress-dependent autophagy, and the specific mechanism has not been further investigated [121].

### Stemness maintenance

Cancer stem cells (CSCs) are responsible for self-renewal, maintenance, and the growth of tumors [122]. The ability of CSCs to evade cell death and metastasis is significant for tumorigenesis [107, 123]. Zinc finger protein X-linked (ZFX), which was highly conserved in vertebrates. According to previous studies, ZFX was concerned with the initiation or progression in various of human cancers [124, 125]. Recently, Lai et al. demonstrated that overexpression of ZFX leaded to the upregulation of Nanog and SOX-2, which could play a vital role in the development of embryonic stem cells (ESCs) [126]. Mechanistically, ZFX could bind to the promoter of Nanog and SOX-2 directly and activate their expression, then contributed to the maintenance of stem-like characteristics of HCC cells [127]. Similarly, ZFX could also upregulate the expression of epithelial cell adhesion molecule (EpCAM) in HCC. Specifically, knocking-down of ZFX could inhibit CSCs-associated gene expression, self-renewal capacity, metastatic potential and tumorigenicity. Depletion of ZFX was demonstrated to reduce the transactivation and nuclear translocation of β-catenin and maintained the stemness of liver CSCs by activating β-catenin. The expression level of ZFX and EpCAM could be a significant prognostic factor of patients in HCC [128].

### EMT and metastasis

The epithelial to mesenchymal transition (EMT) plays a meaningful role in the early steps of metastasis of tumor [104]. In a previous study, TGF-β had been identified as one of the most potential inducer of EMT, which could play a dual role by performing an anti-oncogenic effect at the early stages of tumor and being oncogenic at later stages [109, 129]. As one of the classic EMT activators, ZEB1 plays a significant role in HCC. Li et al. suggested that ZEB1 markedly enhanced the Wnt signaling pathway, promoted the proliferation and migration of HCC, and this phenomenon could be abolished by miR-708 [130]. This regulatory mechanism might provide a new therapeutic target for the treatment of HCC. In addition, Snail, another classic transcription factor, is also involved in the induction of EMT. For example, Jiao et al. demonstrated that Snail and E-cadherin are negatively correlated with mRNA and protein levels in HCC cells, and the high expression level of Snail in HCC often indicates a poor prognosis [131]. Recently, Wang et al. revealed that ZNF703 overexpression could promote HCC metastasis and sorafenib resistance by regulating EMT via upregulating CLDN4. Precisely, ZNF703 activated CLDN4 by binding directly to promoter of CLDN4. Moreover, the Kaplan–Meier analysis showed that ZNF703 could be considered as an indicator for predicting the prognosis of patients with HCC [106]. GATA4 was shown as a ZFP that was identified as a regulator of cardiac development and adult cardiac hypertrophy [132]. Overexpression of GATA4 could lead to the upregulation of E-cadherin and the downregulation of N-cadherin and vimentin, which were vital markers in the EMT process. This phenomenon could result in the mesenchymal-to-epithelial transition of HCC cells, but the specific mechanism was still unknown [133]. ZNF503 was reported to function as a transcriptional repressor in breast cancer and increase mammary epithelial cell proliferation [112, 134]. Recently, the role of ZNF503 in the development of HCC and tumor initiation was uncovered. Yin et al. suggested that mRNA level and protein level of ZNF503 were upregulated in HCC tissues and cell lines. Additionally, ZNF503 could promotes invasion, migration, and EMT processes in HCC. Mechanistically, ZNF503 was demonstrated to be recruited to promoter of GATA4, then represses its expression, which played a reverse role of ZNF503 [135].

### Metabolism reprogramming and glucose metabolism

Cellular metabolism functions as a flexible network not only in normal tissues but also in the development of malignancies [136]. Metabolic reprogramming is the process that tumor cells reprogram the acquisition and metabolism of nutrients to meet their needs for energy, protein synthesis, and maintenance of redox homeostasis [137]. In the past decades, ZFP91 had been reported to be associated with inflammatory response but rarely studied in the metabolism reprogramming [138]. Recently, Chen et al. demonstrated that ZFP91 could inhibit hnRNP A1-dependent PKM splicing and ultimately suppress glucose metabolism reprogramming, cell proliferation and metastasis of HCC. Specifically, overexpression of ZFP91 could promote the Lys48-linked ubiquitination of hnRNP A1 at lysine 8 and proteasomal degradation, which could block the process of hnRNP A1-dependent PKM splicing, and result in the downregulation of PKM2 (Pyruvate kinase M2) and the upregulation of PKM1 (Pyruvate kinase M1) [139]. The formation of PKM2 is critical in the Warburg effect in cancer cells [108]. The Warburg effect causes an increase in glucose uptake and lactate production, which provides a selective advantage for tumor progression [140]. From the perspective of treatment, blocking up the ZFP91-hnRNP A1 pathways may be an effective method in the future.

A20, also known as tumor necrosis factorα-induced protein 3 (TNFAIP3), is an E3 ubiquitin ligase containing ring finger domains and is also a hotpot in immunoregulation [113, 141, 142]. Recently, A20 was found to correlate with glucose metabolism. Feng et al. revealed that A20 could interact with PFKL (phosphofructokinase, liver type) and promote its ubiquitination and degradation, thus inhibiting glycolysis in HCC cell lines and ultimately inhibiting proliferation, migration, and glycolysis of HCC [143]. PFK (phosphofructokinase) was found as the most important rate-limiting enzyme in glycolysis, and PFKL was one of its isoforms in the human liver [144, 145]. This study filled the gap in the glycolytic pathway and provided possible therapeutic targets for HCC treatment.

### Regulation of inflammation

Liver is the main organ where immune response and immune tolerance occur, and many immune reactions in normal hepatocytes and HCC cells may impact on the immune function of the liver [146]. Recently, several researches showed that ZFPs could influence important process of immune responses. Fortunately, Zhang et al. suggested that the ZFP Miz1 could suppress liver tumorigenesis by restraining hepatocyte-driven macrophage activation and inflammation [105, 147]. On the one hand, the cytosolic Miz1 could interfere with the interaction between MTDH and RelA through competitive binding, thereby limiting DEN/ccl4-induced activation of NF-kB in hepatocytes in HCC. Additionally, Miz1 could inhibit MTDH phosphorylation by IKK (the IkB kinase complex), which was an activator of NF-kB after activated by varies extracellular stimuli, then inhibiting NF-kB transcriptional activity [111]. On the other hand, hepatocyte-specific Miz1 decrease produced a unique hepatocytes subset with the up-regulation level of TNF-α, IL-1b, IL-6, and CCL4, which promoted tumor-infiltrating macrophages to a pro-inflammatory phenotype, thereby promoting an inflammatory response in HCC.

## ZNF191: double-edged sword in HCC

**Fig. 3:**
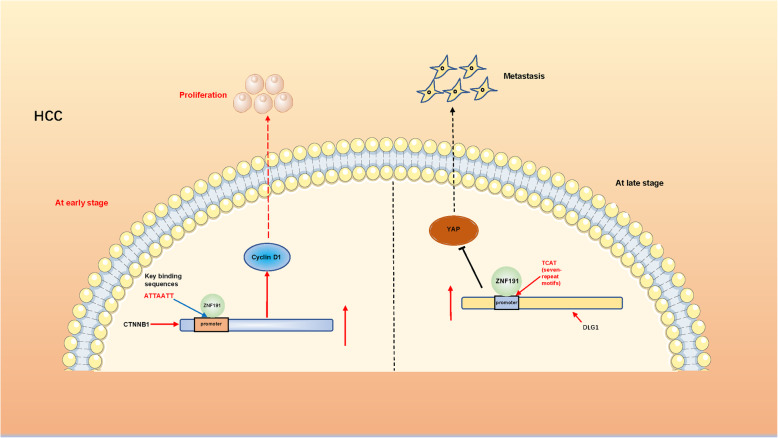
ZNF191 plays an opposite role in different stages of hepatocellular carcinoma. At early stage, ZNF191 activated the expression of CTNNB1 and its downstream gene Cyclin D1 by binding to the promoter region of CTNNB1, ultimately promoted the proliferation of HCC. At late stage, ZNF191 activated its expression by binding to the promoter of DLG1, thereby inhibited the activation of YAP and the migration of HCC cells, eventually inhibited the metastasis of HCC

In most conditions, ZFPs tend to play only one of the two effects, anti-oncogenic or oncogenic. However, during the past few years, some studies have shown that one ZFP may play both oncogenic and tumor-suppressive roles in HCC (Fig. 3). ZNF191, also known as ZNF24, belongs to the scan domain family’s transcription factors containing Krüppel-like zinc finger. It includes four zinc finger motifs that can encode potential DNA binding regions [148]. As a transcription factor that recognizes the TCAT motif explicitly, it plays an essential role in mammalian development, especially during the embryonic period. Within other areas of physiological function, it has been reported to promote the migration of endothelial cells and vascular smooth muscle cells and even promote DNA replication [148–150]. In HCC, ZNF191 was initially reported to enhance the transcriptional activity of β-catenin by binding to nucleotides located at − 1254/− 1224 on its promoter region, thereby upregulated the expression of its downstream target gene cyclinD1 and ultimately promoting the proliferation of HCC cells [151]. A few years later, the same team’s research about ZNF191 in HCC emerged, and this time it was playing an adverse role. Wu et al. revealed that ZNF191 activated DLG1 expression by directly binding to the DLG1 promoter through its typical TCAT repeat sequence, subsequently inhibiting the migration and YAP activation of HCC cells and ultimately the metastasis of HCC [152]. Recently, another study revealed that ZNF191 could activate the WNT signaling pathway through transcriptional upregulation of Wnt8B, which ultimately promoted the proliferation of HCC. Like β-catenin, the promoter region of Wnt8B was bound by ZNF191, which in turn enhanced Wnt8B transcriptional activity, thereby activating the Wnt signaling pathway [153]. It was worth noting that the two mechanisms could co-exist in HCC, which exerted both promotion and inhibition functions. Consequently, the expression level of ZNF191 and associated disease background must be clarified before targeting therapy for ZNF191-associated HCC, and the coexistence of these two mechanisms in the context of ZNF191 also deserves further investigation. We believe that ZNF191 has the potential to be a prognostic factor for HCC and a key therapeutic target as research progresses.

## Conclusion and perspectives

As one of the largest transcription factor families in the human genome, ZFPs play diverse roles in cell biological functions, such as cell differentiation, apoptosis, transcriptional regulation, cell metabolism, immune response. In the past decades, there have been more reports about the role of ZFP in cancer, especially in HCC. HCC is still one of the cancers with the highest mortality rate in the world with many unsolved problems for early diagnosis and postoperative treatment. In this review, we described the role and related mechanisms of ZFPs in the development of HCC.

Above all, zinc finger motifs have different types, including Cys2His2 (C2H2) like, Gag knuckle, Treble clef, Zinc ribbon, Zn2/Cys6, TAZ2 domain like, Zinc binding loops and Metallothionein. The C2H2 zinc finger motif is the largest of all zinc finger motifs. In the C2H2 motif ZFP, in addition to the zinc finger structure, also contains other common domains, such as the KRAB domain, the BTB domain, SCAN domain and SET domain. These domains could bind to DNA, RNA or proteins to function. Secondly, ZFPs were also reported in regulating post-transcriptional modification and protein-protein interaction. Finally, ZFPs affect the development of HCC in different processes, such as cell cycle, apoptosis and even metabolism. Among them, ZNF191 shows a distinct effect in the early and late stages of HCC. Some ZFPs have also been reported as prognostic factors in HCC. For example, the high expression of ZBTB20 and ZNF689 in HCC patients was strongly associated with poor clinical prognosis as well as high recurrence rates [154, 155]. In a latest study, Sun et al. revealed that zinc finger protein 2 gene (ZIC2) was able to predict the prognosis of HCC, and ZIC2 was positively correlated with immune infiltration cells in HCC patients [156]. Therefore, due to the different roles that ZFPs play in HCC, it is possible to invent inhibitors for a specific ZFP or to interfere with the expression of its target gene, and these approaches may provide new thinking about ZFPs in HCC and even in other cancers.

In a word, ZFPs play an important role in tumorigenesis of HCC, and more mechanisms need to be further studied. The targeted drugs of ZFPs in HCC need to be further explored.

## Data Availability

Not applicable.

## Abbreviations

ZNF: zinc finger
HCC: hepatocellular carcinoma
HBV: hepatitis B virus
HCV: hepatitis C virus
ZFP: zinc finger protein
TF: transcription factor
C2H2: cys2his2
BTB: Broad-complex, Tramtrack, and Bric-a-brac
KRAB: Krüppel-associated box
SCAN: SRE-ZBP, CTfin51, AW-1 and Number 18 cDNA
POZ: poxvirus and zinc finger
ZSCAN: zinc finger and scan
KZFPs: Krüppel-associated box domain-containing zinc finger proteins
TEs: transposable elements
ES: embryonic stem
Trx: trithorax
SAM: S-adenosyl-L-methionine
ZEB1: zinc finger E-box binding homeobox 1
HDGF: hepatoma-derived growth factor
EC: endometrial carcinoma
SMRZ: striated muscle ring zinc finger protein
HSPCs: hematopoietic stem and progenitor cells
PGC-1β: proliferator-activated receptor gamma coactivator-1β
HIF-1α: hypoxia-inducible factor-1α
DNL: de novo lipogenesis
FLD: fatty liver disease
HPCs: hematopoietic progenitor cells
SSC: skeletal stem/stromal cell
GSCs: glioma stem cells
Gfi1: growth factor independent 1 transcriptional repressor
LPS: lipopolysaccharide
TLR: toll-like receptor
ZFYVE1: zinc finger FYVE-type containing 1
TLR3: toll-like receptor 3
IL-17R: IL-17 receptor
ER: endoplasmic reticulum
CDKs: cyclin-dependent kinases
CKIs: cyclin-dependent kinase inhibitors
CDC6: cell division cycle 6
MDIG: mineral dust-induced gene
DISC: death-inducing signaling complex
FADD: fas-associated protein with death domain
Bax: bcl2 associated x
Bcl-2: bcl2 apoptosis regulator
PCAF: p300/cbp-associated factor
CSCs: cancer stem cells
ZFX: zinc finger protein x-linked
ESCs: embryonic stem cells
EpCAM: epithelial cell adhesion molecule
EMT: epithelial to mesenchymal transition
PKM2: pyruvate kinase m2
PKM1: pyruvate kinase m1
TNFAIP3: tumor necrosis factorα-induced protein 3
PFKL: phosphofructokinase, liver type
PFK: phosphofructokinase
IKK: the IkB kinase complex
